# Omics Driven Understanding of the Intestines of Parasitic Nematodes

**DOI:** 10.3389/fgene.2019.00652

**Published:** 2019-07-25

**Authors:** Douglas P. Jasmer, Bruce A. Rosa, Rahul Tyagi, Makedonka Mitreva

**Affiliations:** ^1^Department of Veterinary Microbiology and Pathology, Washington State University, Pullman, WA, United States; ^2^McDonnell Genome Institute, Washington University, St. Louis, St. Louis, MI, United States; ^3^Department of Internal Medicine, Washington University School of Medicine, St. Louis, MI, United States

**Keywords:** nematode, intestine, genome, transcriptome, proteome, miRNA, dsRNA

## Abstract

The biological and molecular complexity of nematodes has impeded research on development of new therapies for treatment and control. We have focused on the versatility of the nematode intestine as a target for new therapies. To that end, it is desirable to establish a broad and deep understanding of the molecular architecture underlying intestinal cell functions at the pan-Nematoda level. Multiomics data were generated to uncover the evolutionary principles underlying both conserved and adaptable features of the nematode intestine. Whole genomes were used to reveal the functional potential of the nematodes, tissue-specific transcriptomes provided a deep assessment of genes that are expressed in the adult nematode intestine, and comparison of selected core species was used to determine a first approximation of the pan-Nematoda intestinal transcriptome. Differentially expressed transcripts were also identified among intestinal regions, with the largest number expressed at significantly higher levels in the anterior region, identifying this region as the most functionally unique compared to middle and posterior regions. Profiling intestinal miRNAs targeting these genes identified the conserved intestinal miRNAs. Proteomics of intestinal cell compartments assigned proteins to several different intestinal cell compartments (intestinal tissue, the integral and peripheral intestinal membranes, and the intestinal lumen). Finally, advanced bioinformatic approaches were used to predict intestinal cell functional categories of seminal importance to parasite survival, which can now be experimentally tested and validated. The data provide the most comprehensive compilation of constitutively and differentially expressed genes, predicted gene regulators, and proteins of the nematode intestine. The information provides knowledge that is essential to understand molecular features of nematode intestinal cells and functions of fundamental importance to the intestine of many, if not all, parasitic nematodes.

## Introduction

Infections caused by parasitic nematodes result in substantial mortality and morbidity in human populations, especially in tropical regions of Africa, Asia, and the Americas (Pullan et al., 2014). Infections by parasitic nematodes also compromise the health and productivity of livestock species on a global basis. The impact on food production most significantly affects human health in underdeveloped regions of the world where small holders of livestock depend on food animals for basic nutrition and commerce (Perry and Grace, 2009). Losses in livestock production directly deplete resources needed for basic nutrition, income, and trade. The negative impact of parasitic nematode infections in humans and animals is reduced by anthelmintic treatments; however, the propensity of these pathogens to acquire resistance to anthelmintics (Kaplan, 2004; Leathwick, 2013) threatens the existing methods to prevent and treat diseases they cause. Hence, there is a continuing and compelling need to identify new approaches, therapeutic targets, and applications to prevent and treat infections by nematode pathogens.

This review summarizes the progress made in the last 15 years on an approach that focuses on a single tissue of importance for survival of parasitic nematodes (the intestine) with a goal of developing an integrated research model that will have pan-Nematoda application for deriving essential and broadly conserved intestinal cell functions of these pathogens. One anticipated application of this knowledge is advancement toward the development of pharmaco- and immunotherapeutics for the treatment, prevention, and control of infections caused by nematode pathogens. Earlier studies have validated the value of the intestine of parasitic nematodes for this purpose (next section), which prompts the need to develop resources that can accelerate research on this tissue. The summarized progress relates to the establishment of a multiomics database approach to identify pan-Nematoda conserved intestinal cell functions, coupled with the development of methodologies that can be used to experimentally dissect those conserved functions with new applications to therapeutics as one end goal.

## The Nematode Intestine as a Target for New Anthelmintic Therapies

### General Considerations

**Figure 1:**
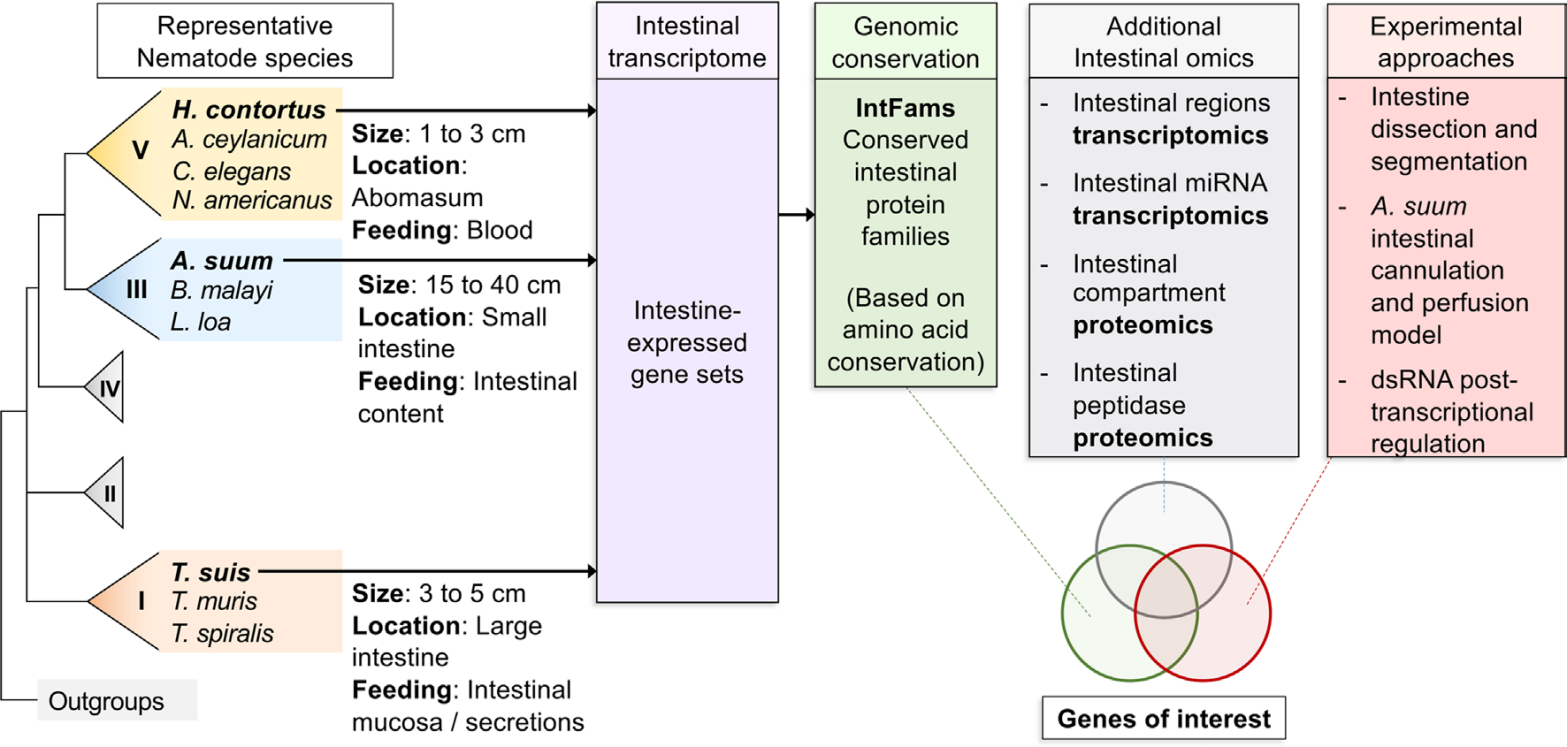
Overall workflow of reviewed intestinal experiments. Transcriptomic and genomic data for core intestinal nematode species are utilized to define intestinal families. Results from additional experiments are intersected and compared to produce prioritized genes of interest to suit experimental needs.

Multiple approaches can be considered when aiming to identify targets for pharmacotherapy, including targets that are expressed in all tissues and parasitic stages and conserved across diverse nematode pathogens (**Figure 1**). However, this requirement overlooks the idiosyncrasies of individual parasite tissues, especially since many, although not all, marketed anthelmintics appear to specifically target one system, the neuromuscular system. Similarly, other tissue/organ systems of nematodes, if disrupted, can theoretically offer the same value for pharmacological targets as the neuromuscular system. The nematode intestine is but one example where experimental data support this view, as will be described in this section. One challenge to more forcefully move forward on tissue-specific research is the relatively shallow knowledge on specific functions, and then molecules, that perform or regulate basic functions in tissues of parasitic nematodes. Furthermore, a focus on an individual tissue does not exclude the possibility that findings generated from one tissue will have application to many or all other tissues and/or stages of parasitic nematodes.

**Figure 2:**
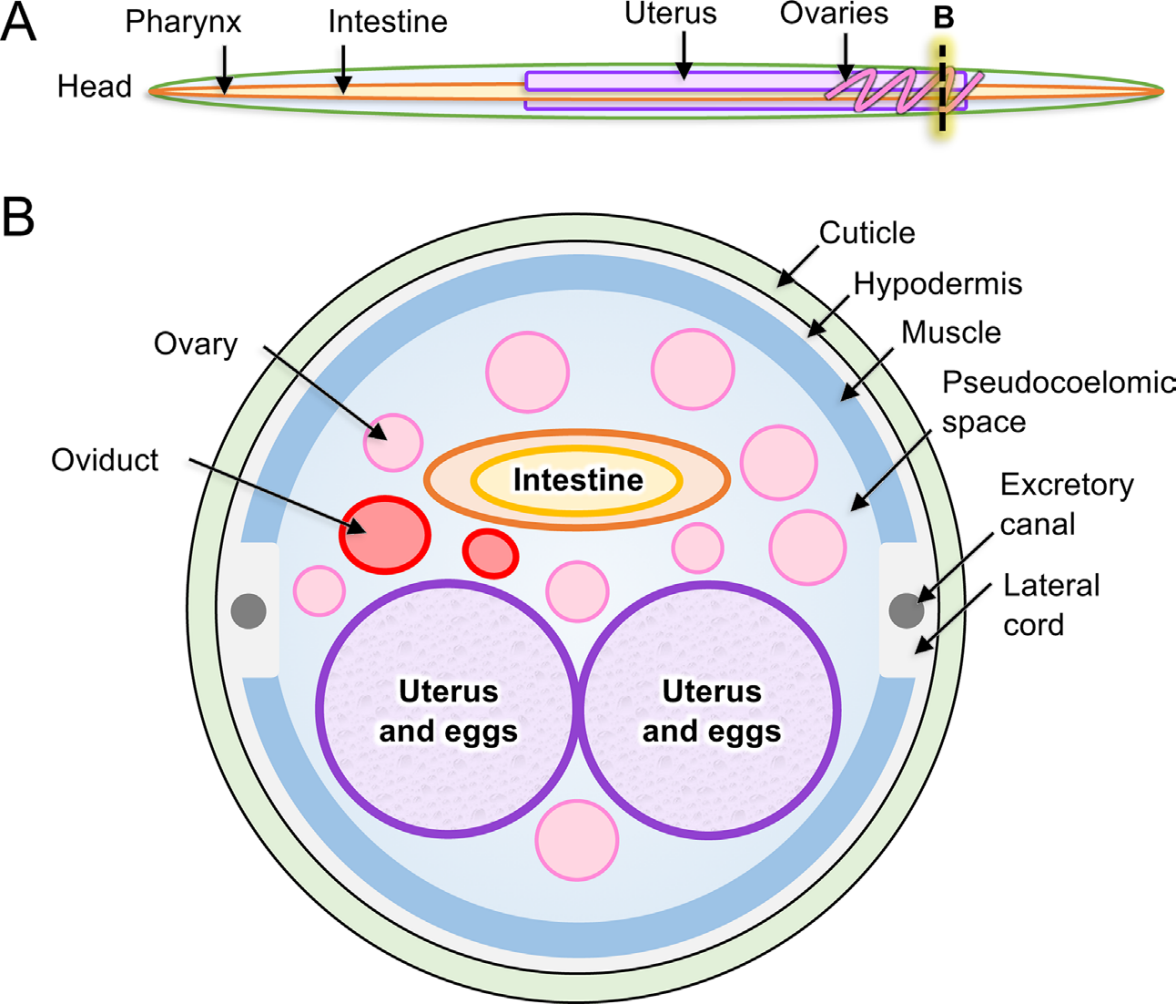
Anatomy of model intestinal nematode species *Ascaris suum*. **(A)** Major organs are identified in an adult *A. suum* female worm, including **(B)** in cross-section.

The tubular intestine of nematodes is composed of polarized epithelial-like cells arranged as a single cell layer (**Figure 2**). Some parasitic nematodes, such as the filarial nematodes, have a somewhat vestigial intestine, possibly due to relocating elements of nutrient acquisition to the cuticular surface (Howells and Chen, 1981). Nevertheless, more recent evidence has clarified roles that intestinal cells of filariae appear to perform (Morris et al., 2015; Ballesteros et al., 2018). The apical intestinal membrane (AIM) of the nematode intestine is continuous with the external environment *via* the nematode stoma and, as such, represents in parasitic species an internal interface with the host. Numerous essential functions are known, or expected, to exist on the AIM related to nutrient digestion and acquisition, general homeostasis of intestinal cells and the intestinal tract, and protection from toxins. Additionally, regeneration of damaged tissue *via* cell proliferation is not known to occur in nematodes. In the absence of effective regeneration, which still requires additional investigation to validate, therapies that cause intestinal cell death and breach of the single cell layer separating the lumen from the pseudocoelom are likely to have lethal outcomes for the parasite. Several lines of research indicate that the intestine of parasitic nematodes has high potential for targeting in immunotherapies and pharmacotherapies that are relevant to treatment, prevention, and control of infections by parasitic nematodes as discussed here.

### Immunotherapeutic Applications of Nematode Intestinal Antigens

From an immunotherapeutic perspective, digestive enzymes localized to the AIM provide one example of antigens for inducing protective immune responses, and antibodies in particular. The expectation is that when the relevant antibodies are ingested by parasitic nematodes, they can inhibit nutrient digestion leading to parasite expulsion. Progress on this approach has been most impressive in blood-feeding parasitic nematodes, such as *Haemonchus contortus* and hookworms, leading to vaccine preparations with potential value (Knox et al., 2003; Knox, 2011; Hotez et al., 2013; Bassetto et al., 2018). However, application of this approach to parasitic nematodes that feed at other host locations has not been sufficiently investigated. For instance, *Ascaris* spp. live in the lumen of the small intestine, and *Trichuris* spp. burrow into the lining of the colon and cecum. Additionally, it remains to be determined if the immune mechanism(s) involved depends on inhibition of functions within the intestinal lumen of the parasite, which has been an underlying premise of this approach. Intestinal antigens from *H. contortus* also localize to the abomasal mucosa and induce local mucosal responses during infection (Jasmer et al., 1996; Jasmer et al., 2007), the importance of which to protective immunity remains undetermined. Despite lack of knowledge of mechanisms involved, intestinal antigens have proven value for inducing immunity against some parasitic nematodes and have likely application to others.

A more recent nuance of possible immunization with intestinal antigens involves exosomes from *Heligmosomoides polygyrus* that are isolated from excretory–secretory products. The exosomes are thought to derive at least in part from the intestine of *H. polygyrus* released in intestinal excretions from the worm (Buck et al., 2014). Immunization with the exosome preparation induced protective immunity against challenge infections in mice (Coakley et al., 2017). It will be of interest to learn if there is a role of intestinal antigens in inducing this immunity.

### Pharmacotherapies That Cause Irreparable Damage to the Nematode Intestine

Effective pharmacotherapy that targets the nematode intestine has previously been demonstrated. The first and most thoroughly investigated examples involve benzimidazole anthelmintics. Benzimidazole treatment of host animals infected with *Ascaris suum*, *H. contortus*, or other parasitic nematodes leads to disruption of intestinal cell architecture and frank disintegration of intestinal tissue; the range of observations depends on species and experimental designs (Borgers and De Nollin, 1975; Borgers et al., 1975; Atkinson et al., 1980; Comley, 1980; Zintz and Frank, 1982; Jasmer et al., 2000; Hanser et al., 2003; O’Neill et al., 2015). The anterior region of the *H. contortus* intestine displayed hypersensitivity regarding these effects, which reflected the occurrence of relatively rapid (within 12 h), irreparable intestinal cell damage, inclusive of nuclear DNA fragmentation, from which the parasite is unlikely to recover (Jasmer et al., 2000). Meanwhile, in *Brugia malayi*, flubendazole exposure does significantly less damage to the intestine, and after a brief exposure *in vitro*, the intestinal and hypodermal damage to adult worms resolved over time after transplantation into the host (O’Neill et al., 2016). The observations clearly show that idiosyncrasies of specific tissues and tissue regions are of importance in the context of inducing irreparable tissue damage, which has high value as an end point for effective anthelmintics. Regarding a possible mechanism, current evidence supports that benzimidazoles inhibit microtubule-mediated apical vesicle transport of hydrolytic enzymes (digestive enzymes) in intestinal cells, which then become dispersed in the cytoplasm where intracytoplasmic digestion of intestinal cells/tissue might occur (Shompole et al., 2002). Stating this possibility in another way, benzimidazoles appear to induce a pathologic process that leads to irreparable intestinal cell damage, which goes beyond simple disruption of nutrient acquisition as might be expected for a downstream effect on intestinal cells by neuromuscular toxins that inhibit pharyngeal pumping. It seems clear that elucidating the actual mechanism responsible will present opportunities to identify alternative methods (targets and drugs) to induce this irreparable damage. If the specific hypothesis is correct, then multiple cellular components of the apical secretory process represent potential targets for inducing this lethal effect in nematode intestinal cells. An additional possibility is that other pathologic processes contribute to, or are fully responsible for, the irreparable damage to intestinal cells. Hence, it will be important to develop methods that can distinguish among these possible explanations. The pan-Nematoda multiomics resource described below can greatly facilitate progress on sorting these alternative explanations and identification of potential new anthelmintic targets.

A second example involves use of suramin against filarial nematodes (Howells et al., 1983) in which the anthelmintic effect was attributed to activity against the intestine, producing ultrastructural changes. Notably, the timeframe for efficacy extended to 5 weeks posttreatment, indicating survival for a prolonged period despite apparent intestinal tissue damage.

A third example of pharmacotherapy targeting of the intestine involves the use of *Bacillus thuringiensis* crystal (CRY) protein toxins. The pore-forming CRY toxins bind to the AIM upon ingestion by insects and nematodes and initiate an irreparable pathologic process that leads to death of the insect or nematode, presumably resulting from generation of pores in the AIM (Hu and Aroian, 2012). This effect also appears (Huffman et al., 2004; Hu and Aroian, 2012) to involve only intestinal cells in the live invertebrates. CRY proteins have also demonstrated interesting utility as anthelmintics for parasitic nematode infections when administered to host animals (Cappello et al., 2006). Once again, it is possible that other inducible pathologic processes contribute to the demise of intestinal cells initiated by CRY toxin. For instance, intestinal factors (p38 MAP kinase pathway) that confer protection against CRY toxins have been identified in *C. elegans*, and better understanding of the protective mechanisms could aid understanding of pathologic mechanisms involved.

A fourth example involves trans-cinnamaldehyde-induced destruction of intestinal tissue in *A. suum* larval stages (Williams et al., 2015). It is worth noting that the pathologic mechanism(s) induced is unclear, and the damage is not restricted to intestinal cells; nonetheless, prior studies have contributed to our knowledge on the susceptibility of intestinal cells in parasitic nematodes to diverse anthelmintic modalities.

### Take-Home Lessons From Past Findings

Major points that can be taken from the foregoing examples include: 1) biological idiosyncrasies (cellular processes specific to the intestine, or accessibility by location on an internal interface with the host) make the intestine of parasitic nematodes a particularly compelling target for immunotherapies and pharmacotherapies; 2) components of the AIM are implicated in roles related to mechanisms of action for at least three of the five (AIM antigens, benzimidazole anthelmintics, and CRY toxins) described immunotherapeutic and pharmacotherapeutic examples, which makes this membrane surface especially interesting; 3) specific processes either involving inhibition of apical secretion or disruption of the AIM have been implicated in pathology induced by benzimidazole anthelmintics and CRY toxin anthelmintics, respectively; and 4) idiosyncrasies of the intestine translate into apparent irreparable tissue damage for at least two of the four (benzimidazoles and CRY toxins) pharmacotherapy examples discussed. The various immunological and pharmacological findings have different conceptual origins (e.g., direct inhibition of digestion and nutrient acquisition, inhibition of apical secretion, or direct disruption of the AIM, respectively), which might lead to an array of new targets to achieve similar outcomes once the mechanisms are better understood for each of them. Hence, there are compelling reasons to improve capabilities for research on intestinal cells in parasitic nematodes, including generation of deeper molecular understanding of nematode intestinal cell functions and developing experimental capabilities to test predictions, progress on both of which are described in the sections below.

## Pan-Nematoda and Multiomics Approach to Research on the Intestine

### General Rationale

Despite most nematodes having a recognizable intestinal tract, the morphology and organization of this tissue are diverse among groups, suggesting functional diversity. At a tissue level, intestinal development ranges from superficial or somewhat vestigial (Eisenback, 1985), to the more common occurrence of a fully developed intestinal tract (Munn and Greenwood, 1984). Nevertheless, intestinal cells reportedly can be organized as syncytia (multinucleate, Munn and Greenwood, 1984), or not, with polyploid nuclei that vary among species from an estimated 4N for adult *A. suum* (Anisimov et al., 1975) to 32N for adult *C. elegans* intestinal cells (Hedgecock and White, 1985). Otherwise, phylogenetic variation of intestinal cells has generally been understudied. More recent progress is summarized in the following sections to clarify basic intestinal cell functions that are conserved at molecular and cellular levels among most nematode species. This progress was facilitated by the selection of core species that span the phylum and represent much of the phylogenetic distance across the Nematoda. The design supported development of a pan-Nematoda multiomics database that was interrogated to investigate broadly conserved biological functions of intestinal cells/tissue for applications toward new pharmacotherapies and immunotherapies.

### Selection of Research Core Species That Sample the Broad Phylogenetic Diversity of the Nematoda

Core adult parasite species have been selected based on four main criteria: 1) each represented a major lineage that collectively represented much of the phylogenetic diversity of the Nematoda and concurrently incorporated the fewest species needed for this purpose; 2) each species could easily be dissected to provide sufficient intestinal tissue for high quality transcriptomic data generation and analyses; 3) extensive genome sequence was available, or near-term obtainable; and 4) each species presented potential for development, or was already established, as an experimental model for research on the intestine. The core species selected (**Table 1**) included: 1) *H. contortus*, a clade V haematophagous parasitic nematode of small ruminants and longtime model for research on its intestine; 2) *A. suum*, a clade III parasitic nematode of the small intestine of swine and humans, the large size of which provided unique advantages that facilitated this research; and 3) *Trichuris suis*, a clade I parasitic nematode of the cecum and large intestine of swine that supported our criteria and provided a needed connection to the most ancient clade of the Nematoda. Each of these core species also has importance for human or veterinary medicine and provides examples of soil-transmitted helminths in their own context. Finally, each also occupies distinct anatomical locations in the host and obtains nutrients from distinct host compartments (blood, intestinal lumen content/epithelial cells, or nutrients accessible from attachment to the mucosa, respectively). Thus, these three core species represent the extensive phylogenetic and biologic diversity sought with this design.

**Table 1:** Genome statistics for core helminth species used to study intestinal functions.

Statistic	*Haemonchus contortus* (Howe et al., 2017)*	*Trichuris suis* (Coghlan et al., 2019)	*Ascaris suum* (original) (Jex et al., 2011)	*Ascaris suum* (improved) (Wang et al., 2017)
Genome size (Mb)	283.4	63.8	272.8	298.0
Coding genes	19,430	9,831	18,542	16,778
Contigs	192	5,498	46,119	1,527
Scaffolds	7	306	29,831	415
BUSCO completeness	92.2%	80.8%	96.4%	96.5%
N50 length (Mb)	47.4	1.3	0.41	4.6
N50 number	3	16	179	21

*Improved version of published genome (Laing et al., 2013).

The multiomics approach delineated the elements of genomes, transcriptomes, proteomes (predicted or/and determined by mass spectrometry), and regulomes (microRNA) that serve intestinal cell functions in a pan-Nematoda context. This effort identified intestinal genes, predicted RNAs, microRNAs, and proteins for which degree of conservation among species was documented to the extent possible. Resulting knowledge bases generated are summarized in the sections below and in **Table 2**.

**Table 2:** Available omics resources useful for the study of the helminth intestine.

Dataset type	Dataset description	Use for intestinal studies
*Genomic*	Genome assembly/annotation for *A. suum* (Jex et al., 2011), *H. contortus* (Laing et al., 2013), and *T. suis* (Wang et al., 2015)	Source for all gene and protein sequence information for core helminth intestine species
	Ortholog matches to other species (Rosa et al., 2014; Wang et al., 2015)	Gene conservation across helminth species/helminth-specific genes
	Wellcome Sanger Institute genome resource (www.sanger.ac.uk/resources/downloads/helminths)	Additional helminth genomes and BLAST database
	Wormbase Parasite (https://parasite.wormbase.org) (Howe et al., 2017)	Helminth genome repository/BLAST services, gene enrichment testing/variant effect predictor
*Genomic functional annotations*	KEGG (Kanehisa et al., 2016)	Biological pathways relevant to intestine
	Gene ontology (Ashburner et al., 2000)	GO terms related to intestine
	MEROPS (peptidases) (Rawlings and Morton, 2008)	Specific classification of digestive peptidases
	Interpro domains (Hunter et al., 2012; Jones et al., 2014)	Additional specific functional annotation
	5’ upstream UTR binding motifs (Rosa et al., 2014)	Identify transcription factors that may modulate expression of intestinal genes
	RNAi phenotype in *C. elegans* (Piano et al., 2000; Piano et al., 2002; Fernandez et al., 2005; Sönnichsen et al., 2005)	Identify intestine-related and lethal/sterile phenotypes
	Protein–protein interactions* (Simonis et al., 2009)	Target intestinal proteins that interact with many other intestinal proteins
*Transcriptomic*	RNA-Seq of 10 *A. suum* tissues (Rosa et al., 2014)	Identify intestine-overexpressed genes in adult male and female *A. suum*
	RNA-Seq of anterior, middle and posterior *A. suum* intestine (Gao et al., 2016)	Identify intestinal genes and miRNAs expressed more highly in various regions of the intestine
	Intestinal RNA-Seq expression from *H. contortus* and *T. suis* (Wang et al., 2015)	Identify intestine-expressed genes in phylogenetically distinct *A. suum* orthologs
*Proteomic*	Proteins detected in various A. suum intestinal compartments (Rosa et al., 2015)	Confident identification of proteins from intestinal tissue, apical and peripheral intestinal membranes, intestinal lumen, and pseudocoelomic fluid
	Peptidases in *A. suum* intestine (Jasmer et al., 2015)	Detailed list of proteomics-confirmed peptidases on the apical intestinal membrane, and in the intestinal lumen
*Multiomics*	Intestinal families (IntFams) (Wang et al., 2015)	Confident nematode-conserved and consistently intestine-expressed genes using multiple species
	Helminth.net online resource (Martin et al., 2015)	A collection of omics databases with tools to search genes and functions, perform BLAST searches, view KEGG pathways, browse variants, and perform multiomics comparisons to identify drug and vaccine targets

*Inferred using the closest significant C. elegans ortholog.

## Antecedents to Deep Pan-Nematoda Intestinal Transcriptomics Database Development

### Genome Resources

Next generation sequencing technologies revolutionized many biomedical research fields including parasitology. As technology was advancing, genomes and predicted proteome sequences for nematode species were accumulating and were subsequently improved over the past two decades. The efforts resulted in generating low coverage genome survey sequences and draft genomes before good quality assemblies and annotations were available. While great progress has been made (Coghlan et al., 2019), most of the available parasitic nematode genomes are draft assemblies with many gaps to be closed and accuracy to be improved (with only a few exceptions, such as *H. contortus* and *O. volvulus*) (Cotton et al., 2016; Laing et al., 2016). Postgenomic applications frequently require comparative genomics on a gene and single nucleotide level, and performing these analyses on draft genomes is inadequate. It is more than ever important to continue efforts on minimizing gene fragmentations, eliminating gene model errors, and resolving collapse of recently duplicated and diverged sequences.

### Early Intestinal Transcriptome Research

Experimental observations listed in the previous section motivated early efforts to expand knowledge of intestinal proteins that are expressed by individual parasitic nematodes (Gao et al., 2016), those that are conserved among nematodes (Wang et al., 2015), and those that differentiate intestinal versus other tissues in nematodes (Rosa et al., 2014). Initial comparisons utilized expressed sequence tag (EST) (Yin et al., 2008) and gene microarray (Wang et al., 2013b) methods, and identified a modest number of predicted intestinal protein families (IntFams) that are orthologs conserved among *H. contortus*, *A. suum*, and *C. elegans*, as well as major differences. One notable difference involved a large family of intestinal cathepsin B-like cysteine proteases expressed by *H. contortus*, whereas a single intestinal family member was detected for *A. suum* and 12 family members have been described for *C. elegans* (Jasmer et al., 2004). This early foundation fostered anticipation that while many intestinal functions may be conserved among nematodes, there are likely many adaptations that differentiate nematodes species, and possibly phylogenetic lines.

Due to its large size, *A. suum* (male, 15–30 cm; female, 20–35 cm) is a particularly good nematode model to study tissue transcriptomics, since the individual tissues can be cleanly and accurately dissected. In the first ever comprehensive tissue-specific RNAseq-based transcriptome studies for any parasitic nematode, comparative transcriptomics was performed on three nonreproductive tissues (head, pharynx, and intestine) in both male and female worms, as well as four reproductive tissues (testis, seminal vesicle, ovary, and uterus) (Rosa et al., 2014). This study identified thousands of genes associated with the different tissues (or combinations of tissues), including 1,387 genes overexpressed in the intestine samples (male or female). Examination of the 5′ untranslated region (UTR) sequences preceding the coding sequences for the intestine-associated genes identified enrichment for the motif that is bound by the transcription factor ELT-2, the predominant transcription factor controlling intestinal development and function in *C. elegans* (McGhee et al., 2009). Intestine-enriched functions included nine “Molecular Function” child terms of “hydrolase activity” (GO:0016787; including “cysteine-type endopeptidase activity”). Gene coexpression networks linked some genes of the highly intestine overexpressed genes to other tissues, including the head-overexpressed FAR-1 ortholog, a proposed anthelmintic target with a crucial role in parasitism (Bradley et al., 2001; Basavaraju et al., 2003). Overall, this study provided a foundation for cataloguing and profiling intestinal expression in *A. suum*, and a gene expression and annotation database was built using its datasets (Rosa et al., 2014).

### Pan-Nematoda Database of Intestine Expressed Gene Transcripts and Predicted Proteins

**Figure 3:**
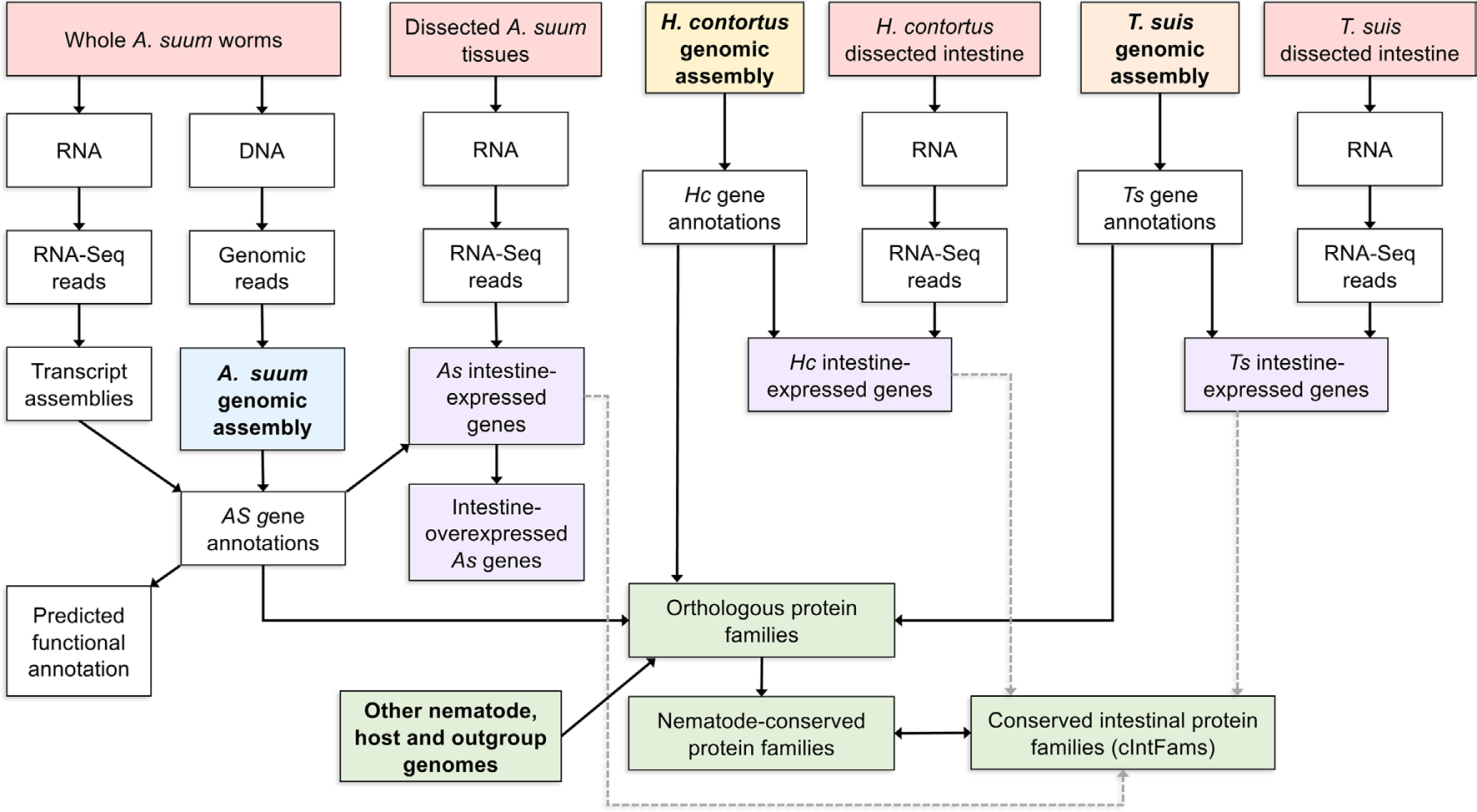
A detailed example of one bioinformatic workflow. Here, a list of conserved intestinal-expressed protein families is produced by intersecting several datasets. The workflow for producing the *Ascaris suum* genome assembly and annotation is shown but omitted for the other two core species.

The next step after the *A. suum* comparative tissue expression data was to determine the level of conservation of intestinal cell functions among nematode lineages and species. Three omics resources were central to this investigation. The first was annotated genomes and predicted proteome information for each of the core species under investigation. The second was deep intestinal transcriptomes (generated by RNAseq) for each of the core species representing clades I, III, and V of the Nematoda. These two resources allowed for direct assessment of intestinal genes/proteins expressed by each of the phylogenetically diverse core species and transcriptomes from other tissues. A third essential omics resource was genome and predicted proteome information from each of the other nematode and outgroup species included in the analysis. As a first step, orthologous protein groups were predicted from combined data for all species (10 nematodes and 5 outgroups) included in the analysis. Then, transcriptomic data from each core species were related to predicted proteome data from each and the orthologous protein groups derived from all 15 species. Intestinal protein families (IntFams) could then be inferred across orthologous protein families identified at any of numerous phylogenetic levels based on the expression from the core species. The relationship of the core species data to inferred intestinal proteomes from other nematode species is shown in **Figure 3**. The study based on intestinal expressed sequence tags identified 241 predicted IntFams from *H. contortus*, *A. suum*, and *C. elegans* (Yin et al., 2008) (based on 3,121 and 1,755 identified transcripts from *A. suum* and *H. contortus*, respectively), whereas the pan-Nematoda RNAseq study (Rosa et al., 2014) expanded the number of IntFams to 10,772 (based on complete gene sets from nematode species spanning the phylum). Phylogenetic assessment of IntFam gene births and deaths among the 10 species analyzed produced phylogenetic tree associations expected for trees of this species assemblage based on other quantitative criteria (Rosa et al., 2014).

The phylogenetic affinities and conservation (predicted orthologs) of the IntFams determined by this analysis were parsed into 14 categories, ranging from IntFams/functions that are conserved among all the core nematode species (**cIntFams**, 2,853 from intestinal transcriptome data) to the ones conserved in all 10 of the diverse nematode species investigated (**nem-cIntfams**, 1,863). One can rationalize the biological or practical importance for each of these groupings, including the taxonomically restricted ones. The most inclusive group for intestinal genes included orthologs conserved in all 10 nematodes and 5 outgroups (**uni-cIntFams**). Conservation of this kind might be viewed to preclude utility of proteins in therapeutic approaches. Nevertheless, insertion/deletion (indel) analysis identified distinctions of potential practical significance between some nematode and mammalian members of these IntFams. Thus, the differences existed below the threshold utilized for parsing orthologous proteins. We can also expect for members of this group that amino acid substitutions may exist that are nemato-centric and have functional significance, such as in the case for many beta tubulins in nematodes (Hoti et al., 2003). Although this particular analysis was not conducted in a comprehensive manner, the database exists to do so. In some cases, IntFam members were detected based on transcripts from each of the core species (cIntFam) but not necessarily from genomes of all nematodes investigated. This level of conservation (clades I, III, and V) based on direct sampling of intestinal transcripts reflects broad conservation across many parasitic nematode species, although maybe not all. Some IntFams lacked representation in all core species (e.g., intestinal transcripts from all core species) but nevertheless had ortholog representation from genomes of all 10 nematode species evaluated. This situation might reflect false negatives (sampling artifact) from intestinal transcriptomes of individual core nematode species, or expression of some intestinal transcripts might be conditional and transient. Consequently, interpretation of the data warrants some caution, and the relationships described should be viewed as a beginning for developing hypotheses rather than a final determination of conservation.

Beyond information that can be gained on the molecular evolution of the intestine in adult parasitic nematodes, we envision multiple uses for this pan-Nematoda database of IntFams. From a basic omics perspective, the information renders less mysterious the specific intestinal proteins, protein isoforms, amino acid sequences, predicted functions, and diversity of those proteins that carry out essential functions of the intestine among nematode species. By organizing the data as predicted orthologs with specific proteins identified from each species investigated, researchers have a template to explore questions of individual interest and with some explicit gene and protein sequence information that can support experimental design efforts. As one example, protein isoforms from multimember families that are differentially expressed in the intestine or other tissues can be prospectively identified, by integrating the IntFam database and the comparative-tissue transcriptome database for *A. suum*, thus resolving substantial complexity ensconced in whole genome and predicted proteome data. Such resolution is critical when the biology of individual tissues is under investigation.

## Increasing Resolution by Studying Intestinal Regions at a Transcriptome and Proteome Level

### Differential Expression of Genes Along the Length of the Intestine and Their Posttranscriptional Regulation

Another dimension of functional dissection involves differential expression of intestinal genes along the length of the intestine. The anterior region of the *H. contortus* intestine is hypersensitive to benzimidazole treatments, by comparison to the posterior region (Jasmer et al., 2000). Consequently, biological differences among intestinal regions exist that appear to have practical applications. To study this in more depth, *A. suum* intestinal regions have been studied using RNAseq approach, resulting in resolving expression differences in a single, comprehensive effort, the results of which will enhance the use of this species in intestinal cell research and likely will have application to other nematode species.

**Figure 4:**
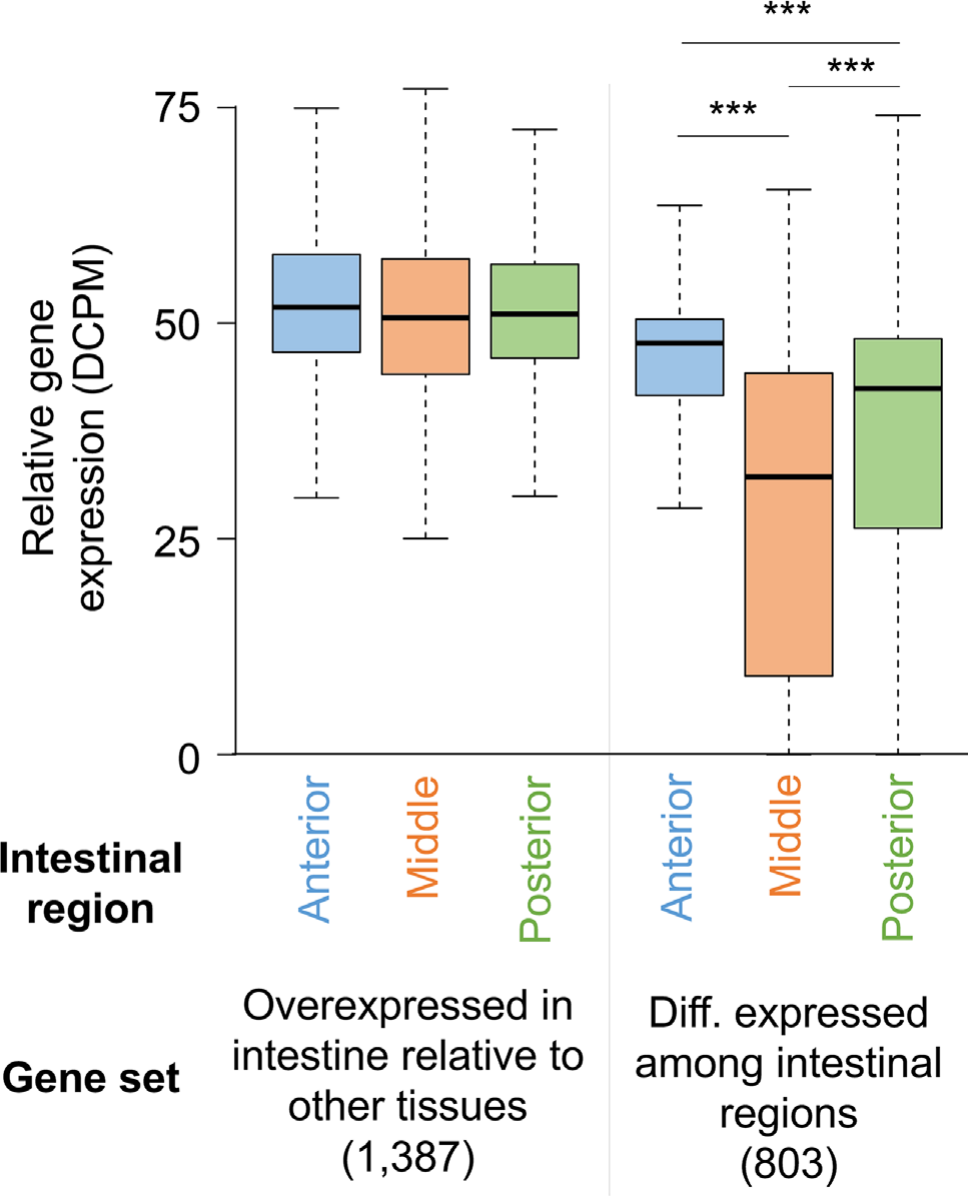
Distribution of gene expression levels for *A. suum* genes in the anterior, middle, and posterior intestine regions among all intestine-overexpressed genes and genes differentially expressed between the regions. ***P < 10^−5^.

Deep sequencing of transcripts obtained from contiguous anterior, middle, and posterior regions of the intestine from male and female *A. suum* worms expanded the set of previously known intestinal genes (Gao et al., 2016). No major differences were identified based on gender, with >80% intestinal genes expressed in both male and female. Genes expressed among the three regions were similar, with only 803 genes being differentially expressed. However, most of these (696/803) had higher expression levels in the anterior region as compared to the middle and posterior regions, supporting that the anterior intestinal region has certain functional distinctiveness as compared to the rest of the intestine (**Figure 4**). This difference was also indicated by assembling the genes based on different expression profiles along the intestinal axis. This less stringent analysis identified genes expressed at higher levels in the anterior region including those encoding certain hydrolases and those with functions related to signal transduction and membrane dynamics. Given that a vast majority of genes were expressed throughout the intestine, although most of these are expressed at relatively low levels, one way of delineating major intestinal functions is to analyze genes with relatively high overall intestinal expression. Based on a comparison with the expression levels of all genes, transcripts for 795 were considered to be highly expressed in the intestine. As expected, some of the functions encoded by these highly abundant intestinal transcripts were related to nutrient uptake and energy metabolism. Other enriched functions included protein synthesis and protein and lipid binding.

Samples from the same regions of the *A. suum* intestine were profiled for miRNAs. This provided another layer of possible differentiation among intestinal regions, i.e., regulators of intestinal gene expression. miRNAs were known to be expressed in gametogenesis and early developmental stages of *A. suum* (Wang et al., 2011). On the other hand, intestinal miRNAs have previously been identified in *C. elegans*, and these were shown to cause effective downregulation of mRNA expression (Kato et al., 2016). We leveraged the availability of samples from different intestinal segments to further expand the knowledge base of nematode miRNAs, their interactions with the transcriptome, and differential expression along the intestinal tract (Gao et al., 2016). As a result of this work, 277 miRNAs were identified in the *A. suum* intestine, and 11 of those were differentially expressed among intestinal regions. To integrate the information from mRNA and miRNA expression sets, potential targets of these miRNAs were predicted for the ∼6,000 genes of the *A. suum* genome that had a 3’UTR sequence available and could be used for miRNA–target prediction. Out of these, 2,063 were predicted to be targeted by at least one intestinal miRNA. As is usually the case with predicted miRNA targets, the predicted miRNA-target network was dense with many-to-many targeting interactions commonly encountered (i.e., multiple targets associated with a given miRNA and vice versa). These predictions need validations, as does the existence of such a highly active regulatory network.

The large number of samples included in this study provided abundance data for both mRNAs and miRNAs that supported a novel statistical approach for predicting miRNA–target associations. Correlations between sample abundances of miRNAs and corresponding predicted mRNA targets identified 503 pairs categorized as most likely to be associated with each other (LAMPs: likely associated miRNA-mRNA pairs). In a similar vein, mean correlations of some miRNAs with corresponding target mRNAs were markedly higher than mean correlations of other miRNAs. These miRNAs were classified as the most likely influential miRNAs (LIMs). Interestingly, encoded proteins of many of the set of predicted targets for the 22 LIMs showed significant functional enrichment [GO (The Gene Ontology, 2019), InterPro (Mitchell et al., 2019), etc.], potentially indicating real biological functions under miRNA regulation. An analysis of miRNA mature and seed sequences identified some miRNAs and miRNA families whose intestinal expression, and hence, potential intestinal function is conserved between *A. suum* and *C. elegans* or *Heligmosomoides bakeri* (Gao et al., 2016). The intestinal miRNA databases offer guidance to determine functions of these regulatory molecules in intestinal gene expression and functions of proteins encoded by target mRNAs.

Resolution of gene expression by transcriptomics along longitudinal regions of the intestine as done with *A. suum* is far more challenging for many other nematodes, parasitic or not. However, integrating information on differentially expressed genes and proteins across *A. suum* intestinal regions with the pan-Nematoda intestinal omics databases can lead to useful predictions on intestinal expression patterns of orthologous genes and proteins from other nematode species.

### Intestinal Functions Detected at a Protein Level

**Figure 5:**
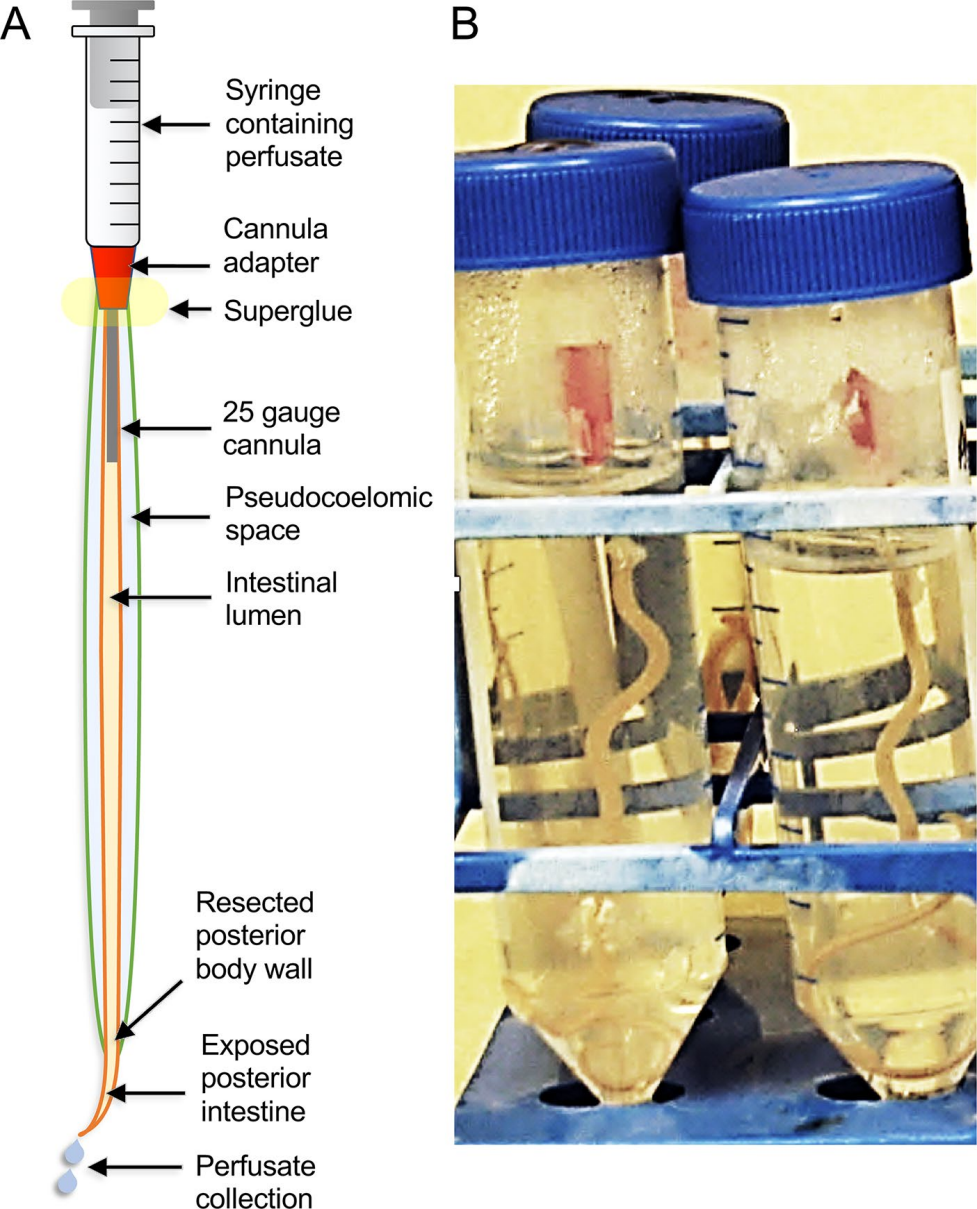
The *A. suum* intestinal cannulation and perfusion system. Shown are **(A)** a diagram description of the system and **(B)** a picture of the actual worms set up in the system, and contained inside of plastic test tubes.

**Figure 6:**
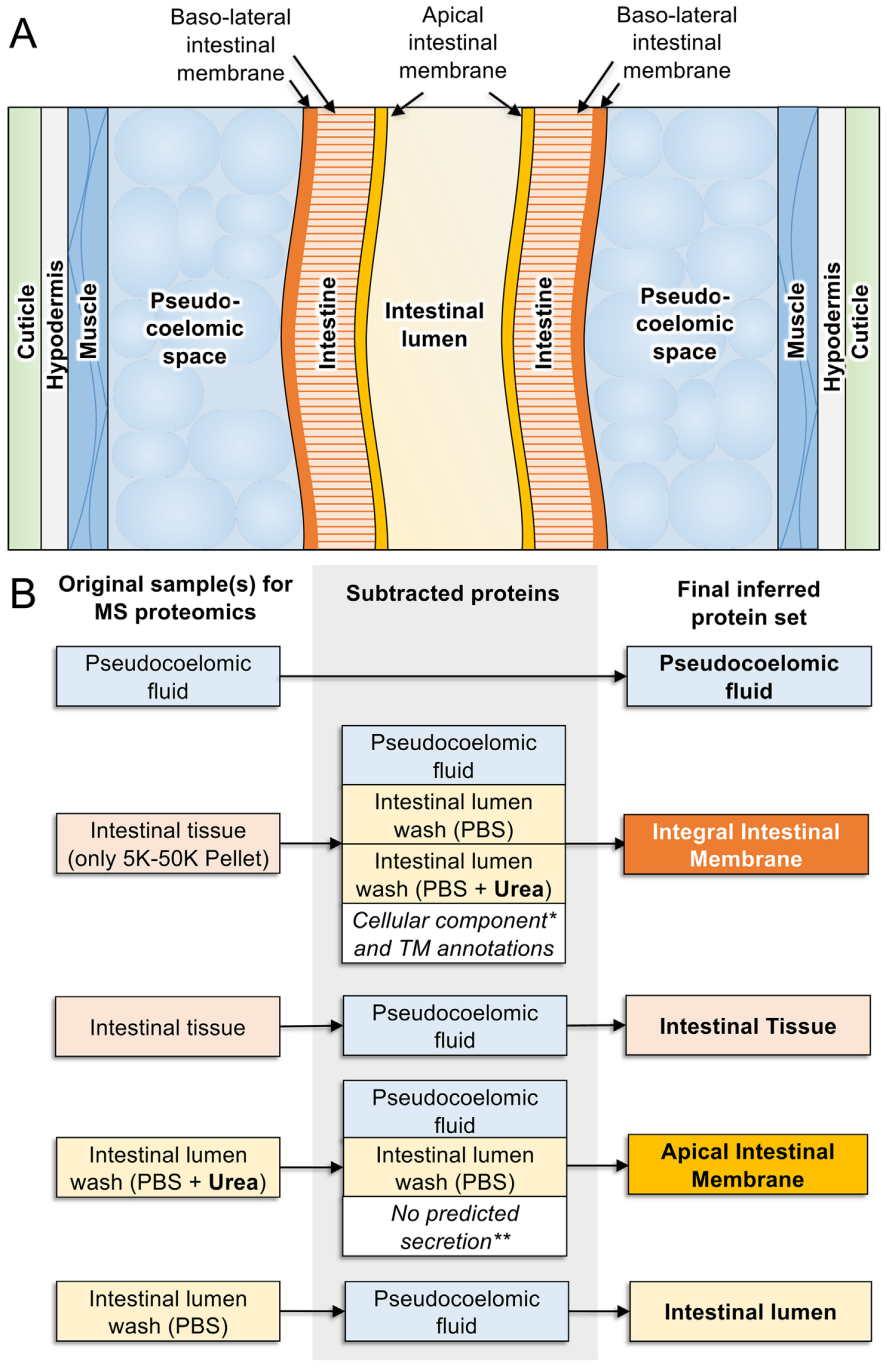
Proteomics-based inference of *A. suum* intestinal proteins in different compartments. **(A)** Anatomy of model intestinal nematode species *Ascaris suum* (transverse section). **(B)** Protein sets detected by MS/MS proteomics from samples harvested from adult *A. suum* worms (left) have other protein sets strategically removed (center) to deduce final protein sets in different intestinal compartments (right). “Integral intestinal membrane” proteins are not labelled as “basal” because they may include some proteins from the apical intestinal membrane as well. *Proteins annotated with “cellular compartment” Gene Ontology terms for endoplasmic reticulum, mitochondria, Golgi apparatus, and nucleus were removed to reduce contamination from proteins embedded in these organelles rather than the external cellular membrane. **Only proteins annotated with predicted classical or nonclassical secretion signals were included since these are better candidates for proteins that are transported to the membrane.

Traditional research on intestinal antigens targeted in vaccine research has identified a modest number of AIM proteins, including many proteases (Andrews et al., 1995; Jones and Hotez, 2002; Smith et al., 2003a; Smith et al., 2003b; Williamson et al., 2003a; Williamson et al., 2003b; Loukas et al., 2004; Williamson et al., 2004; Jasmer et al., 2007; Knox, 2011). Factors that hindered more comprehensive identification of AIM functions include the relative narrow focus of those investigations, available methods, and limitations posed by the nematodes investigated. In contrast, more recent studies took advantage of the large size of *A. suum*, which when coupled with extensive databases of intestinal genes/predicted proteins, supported a proteomic approach that greatly increased knowledge of proteins and predicted functions on the AIM and other cellular compartments of the intestine. Cannulation of the adult *A. suum* intestine (**Figure 5**), below the pharynx, with ordinary blunt-ended hypodermic needles (Rosa et al., 2015) facilitated the perfusion of the intestine with, in this case, phosphate-buffered saline (PBS) to directly collect lumen content and directly identify proteins in this perfusate by mass spectrometry (**Figure 6**). Intestinal antigen research in *H. contortus* (Jasmer et al., 2007) indicated that many of the intestinal proteases are associated with the AIM (Williamson et al., 2003b), but because those proteins had predicted signal peptides and most often lacked evidence of transmembrane regions, it was suggested that they function as peripheral membrane proteins. Thus, *A. suum* intestinal lumen was also perfused with 4 M urea (4 MU) to solubilize peripheral membrane proteins. Additionally, the glycans on many of the *H. contortus* AIM proteases have figured centrally in methods used to isolate, identify, and functionally characterize these proteins (Jasmer et al., 1993; Smith et al., 1993; Smith et al., 1994; Rosa et al., 2015). Similarly, the lectin concanavalin A predominately bound to the AIM in *A. suum* intestinal tissue and was used to isolate and characterize apparent AIM glycoproteins by lectin affinity chromatography. These analyses of intestinal perfusates, lectin binding fractions, additional intestinal membrane fractions, and whole intestinal lysates by mass spectrometry have identify over 1,000 intestinal proteins that were assigned to cellular/tissue compartments based on methods of generating each fraction, and predicted physical properties including, charge, signal peptides, and transmembrane regions.

This approach identified numerous proteins located in the *A. suum* lumen or on the AIM along with predicted functions of those proteins (Rosa et al., 2015). Experimental evidence was also gained on a range of proteases and related activities that were detected in perfusates of the intestinal lumen (Jasmer et al., 2015). About 157 distinct proteins were obtained in the PBS and 4 MU perfusates. Two major groups of proteins attracted the greatest interest in these fractions, digestive hydrolases and channel/transport proteins, which are expected to contribute to ion and nutrient transport. The hydrolases included proteases (28 in 5 classes of proteases) with metallopeptidases being best represented, including 16 different metallopeptidases. Aspartic proteases and serine carboxypeptidases were the next best represented with five and four proteins, respectively, followed by cysteine and threonine proteases representing the remainder. The relative abundance and diversity of apparent proteases identified in these fractions answered questions regarding the comparative need for protein digestion in *A. suum* which lives in the host small intestine where host protein digestion is nearing completion. Apparently, *A. suum* has substantial need for digestion of proteins consumed in this host location. O-Glycosyl hydrolases comprised a second group of hydrolases, which likely contribute to saccharidase activity previously investigated in *A. suum* (Gentner and Castro, 1974), and some of these hydrolases were identified in previous investigations on *A. suum* larval stages and intestinal tissue (Wang et al., 2013a). A third group of hydrolases included several lipases, thus rounding out a basic set of digestive enzymes in the lumen of *A. suum* intestine. The study detected many other predicted functions that fall outside these groups. These collective data can help formulate ideas to target digestive processes in anthelmintic strategies against *A. suum* and possibly other parasitic nematodes.

The detected channel/transport proteins that mediate transport of ions and nutrients across intestinal membranes are of interest because interference with these functions has potential for anthelmintic approaches, and new information about them should facilitate development of these approaches. Information in this context was largely derived from the 4 MU perfusates and the membrane preparation, which included many proteins detected in the 4 MU perfusate as a subset. Over 100 proteins were classified as channel/transport proteins, with predicted transmembrane (integral membrane proteins) or other annotation supporting this assignment, and detection in membrane enriched fractions added further support. However, many of the channel/transport proteins were detected only in the membrane fraction, which does not resolve apical, basolateral, or intracellular membrane localization in intact intestine, although an attempt was made to exclude proteins associated with cytoplasmic organelles. Distinguishing membrane location is important particularly if antibody-mediated inhibition is a goal in vaccine research, as this would likely require localization to the AIM. Accessibility by localization to the AIM may also be an important factor for pharmacotherapy, which becomes much more feasible with knowledge of potential targets that are accessible on the AIM. An example here is the apical localization of the receptor for CRY protein toxins, the absence of which on the AIM is likely to render CRY proteins less effective (Griffitts et al., 2003; Williamson et al., 2003b). Nevertheless, for a large number of predicted integral membrane proteins identified, channel/transport proteins and others, the actual membrane where they reside remains to be determined. Alternatively, some of these membrane proteins were also, or exclusively, detected in the 4 MU fraction, which provided evidence for an AIM localization, although not to the exclusion of the basolateral intestinal membrane (BIM), also. One example highlighted by this research involves subunits of the vacuolar ATPase V1 domain. Seven of the eight subunits were detected in the 4 MU fractions, which likely reflects solubilization by 4 MU (Rosa et al., 2015). The results provide evidence that similar to *C. elegans* (Allman et al., 2009), V-ATPases occupy the AIM and contribute to H+ transport across this surface in *A. suum*. This kind of evidence was obtained for 57 other channel/transport proteins, nevertheless leaving the majority of the predicted integral membrane proteins with little direct evidence for the membrane (s) on which they function.

Examining the sequence conservation of the orthologous proteins detected in different cellular compartments across species spanning the phylum Nematoda revealed significant variability. Most variable were the proteins detected in the intestinal lumen (IL), and integral intestinal membrane proteins were the most conserved among nematodes. Concurrently, proteins homologous to most of the *A. suum* IL proteins were detected in other nematode species, and because hydrolytic enzymes constitute a substantial subset of IL proteins, the result may reflect diversification from common ancestral digestive enzymes among species. Although neutral evolution could account for diversification in this background, directional evolution might be expected if adaptations are required of digestive enzymes for a given species to best exploit nutrients presented by different host niches. Hemoglobinases from hookworms may exemplify this idea in that human hemoglobin and serum proteins were digested more efficiently than orthologous proteins from dogs by the hemoglobinase Na-APR-2 from the hookworm of humans, *Necator americanus* (Williamson et al., 2003a). Despite the lower level conservation for individual protease proteins, major classes of IL proteases (aspartic, cysteine, metallo-, and serine) appear to be conserved among many nematode species and appear to retain broad functional characteristics related to inhibitors of the protease classes, which may have pharmacotherapeutic applications. The omics data utilized to identify probable IL proteins from other nematodes may have application toward identification of IL proteins from other nematode pathogens. It is important to note that many of the predicted proteases that colocalized to the PBS and 4 MU fractions were classified as IL proteins, even though they may function as peripheral membrane proteins that then are released into the lumen.

The proteomics analysis summarized here significantly clarifies functions that might be sited in the various intestinal cell and tissue compartments, and available evidence can be consulted for prospective development of research hypotheses stimulated by information in this resource. As one example, much of the past vaccine research has focused on proteases and their inhibition by host neutralizing antibodies ingested by the parasite. Alternatively, channel/transport proteins sited on the AIM have at least as much attraction as hydrolases for antibody-mediated inhibition of nutrient acquisition and other homeostatic functions (such as ion transport). The numerous specific examples of prospective AIM channel/transport proteins can now be prioritized for investigation in this direction. Additional membrane proteins were identified, some of which likely function on the AIM and can be investigated individually. However, and despite the progress reported, with only about 1,000 intestinal proteins identified by mass spectrometry, the advances are modest by comparison to what can be achieved with *A. suum* in a more concerted proteomic analyses of nematode intestinal cell compartments. Not many nematode species can support the methods and tissue demands needed to gain the insight offered on this topic by the *A. suum* intestinal system. Given that the tools exist to place resulting information into a pan-Nematoda context, this area should be exploited to the full extent that the *A. suum* system can support.

We provide here an example of one of the many ways the generated multiomics database can be interrogated. Published reports provide supplementary tables in which the data have been integrated into a database that includes (for every documented *A. suum* gene) functional annotation, pan-Nematoda phylogeny, RNAseq expression and differential expression, and proteomic data spanning all of the available datasets (**Table 2**) from the publications discussed in this review. This resource allows for straightforward and convenient cross-study dataset comparisons and the prioritization of genes of interest to suit the goals of new studies.

In addition to our ongoing *A. suum* intestinal omics research, other nematode intestinal omics datasets are available in the literature including a transcriptomics study of the *Ancylostoma ceylanicum* intestine, a proteomic study comparing the intestine, body wall, and reproductive tissues of *B. malayi*, and a proteomic study of larval-stage *A. suum* excretory–secretory (ES) products that provides more information about potential intestine-produced products during the larval stage (Wang et al., 2013a). As we have performed for our projects, future research will benefit from intersecting the results from these valuable intestinal omics resources, to provide more information about genes of interest and better insights into conserved and specific intestinal functions.

## Advances on Methods to Experimentally Manipulate *A. suum* Intestinal Cells

### The Intestinal Cannulation and Perfusion System

As with many aspects of helminth research, the small size of many nematodes, limited access to live parasite tissues of interest, and poor *in vitro* survival of the life cycle stages of interest all present challenges for investigations on intestinal cells. Again, *A. suum* presents distinct advantages relative to obtaining abundant biological materials for biochemical analyses. Its large size has facilitated improvements to experimentally investigate intestinal cell functions, particularly as relates to cannulation and perfusion of the intestine (**Figure 5**). This technique supports delivery of controlled treatments (amount, timing) into the lumen for experimental manipulation of the AIM and other intestinal cell functions. Delivery *via* perfusion leads to the immediate achievement of treatment concentrations in the lumen. In contrast, delivery by feeding is variable and difficult to quantify or is unachievable for many parasitic species. Maintenance of the cannulated intestine *in situ* where it is bathed by pseudocoelomic fluid leads to expectations for faithful replication of *in vivo* functions in this setup, particularly over short-term experiments. Cannulated *A. suum* can be maintained in culture media for at least 4 days after which time worms are still motile, although stable maintenance of intestinal cell functions during this time period requires verification. Experimental treatments can also be injected into the pseudocoelom for delivery across the BIM. The advantage of BIM delivery is that pseudocoelomic injections can be done with intact worms, which can then be maintained for several days in *in vitro* culture. Pseudocoelomic delivery of bacteria was shown to induce expression of intestinal transcripts encoding *A. suum* antibacterial factors (ASABF) (Pillai et al., 2003) as one example. However, in many cases, transport across the AIM is not expected to be replicated by the BIM, and transporters may have different polarities if found in both the AIM and BIM. Because BIM delivery of experimental treatments to intestinal cells is not an obvious alternative to dissect AIM functions, simple methods like cannulation and perfusion of the intestinal lumen represent valuable capabilities to investigate functions peculiar to the AIM. It is also likely that the *A. suum* system will support experimental dissection of functions on the BIM, about which even less is known. Nevertheless, double-stranded RNA (dsRNA) treatments delivered by both routes causes knockdown of target intestinal cell transcripts, as discussed below. Hence, the two different routes of delivery may have complementary characteristics for at least some treatments, as described in the next paragraph.

### Manipulation of Intestinal Cell Transcripts by Perfusion of dsRNA

The ability of the intestinal perfusion method to support experimental manipulation of intestinal cell functions has been tested using dsRNA as the treatment for several *A. suum* intestinal genes (Rosa et al., 2017). The genes used in this study were selected based on a number of criteria, including evidence of results with dsRNA from orthologous genes in heterologous species. A series of experiments was conducted in which dsRNA constructs were tested for each of five genes. Based on the results, dsRNA was selected for one of these genes (GS_08011) to assess treatment variables, including dsRNA construct (intact long or dsRNA fragmented by ribonuclease III), amount, and comparative effectiveness of delivery across the AIM or BIM. These studies showed that while knockdown of transcripts was impressive for some genes (up to 99.7% expression reduction), there was also some variability among genes, which is not unusual for *A. suum* or other parasitic nematodes (Selkirk et al., 2012). Perfused quantities down to 0.5 µg per worm caused effective knockdown of transcripts, and knockdown was achieved to approximately the same level in anterior and posterior halves of the intestine. Likewise, knockdown of intestinal transcripts was achieved to approximately the same level for dsRNA perfused into the intestinal lumen or injected into the pseudocoelom (the effect of which was previously investigated for pseudocoelomic delivery of dsRNA) (McCoy et al., 2015). These effects were achieved within a 24-h treatment period.

### Assessment of Off-Target dsRNA Effects

It is important to evaluate off-target dsRNA effects by RNAseq and challenges they might present for interpretation of experimental results even when effectiveness of dsRNA delivery and knockdown of intestinal cell transcripts is established. The *A. suum* dsRNA perfusion study (Rosa et al., 2017) quantified global transcript effects for treatments of dsRNA constructs from two different genes, then control decapitated worms at time 0 or cannulated for 24 h, and anterior and posterior regions of worms from each treatment group. By comparison to other species/model systems, only modest effects were observed for up- or downregulation of off-target genes related to dsRNA treatments. For instance, transcripts for only eight and four intestinal genes showed significant up- or downregulation, respectively, by both dsRNA gene constructs in both anterior and posterior intestinal regions (Rosa et al., 2017). Additional, but still modest, effects were observed related to individual dsRNA gene treatments and then anterior and posterior intestinal regions. Overall, off-target effects will require attention in relation to functional studies, perhaps in the form of using multiple distinct dsRNA constructs to rule out construct-specific effects and incorporating results from complementary approaches for functional analyses.

### Summary of Progress on the *A. suum* Intestinal Research Model

Attempts to manipulate *A. suum* intestinal cells using the perfusion system have been successful. Overall, the published results indicate that the system provides a relatively facile method to manipulate intestinal cell functions, in this case knockdown of target transcripts by dsRNA. Although concomitant knockdown of protein will be an important follow-up to assess the utility of the dsRNA approach to determine protein functions, the research demonstrated the ability of the system to support exogenous manipulation of intestinal cells. The intestinal perfusion system should enable many questions to be investigated regarding functions that reside in the lumen, on the AIM, and in intestinal cells, thus adding motivation to gain a more complete picture of compartments occupied by proteins expressed by intestinal cells. In context of omics resources for the *A. suum* intestinal model coupled with pan-Nematoda IntFam resources, *A. suum* offers valuable attributes to a cooperative multispecies model aimed to derive essential functions of intestinal cells common to many parasitic nematodes. It remains to be determined how the other two core species (*H. contortus* and *T. suis*) will add to the cooperative model. Concomitantly, one can now predict intestinal genes in other parasitic nematodes, and *Strongyloides* spp. provide parasite examples where transgenic methods can be used to test functional predictions *in vivo* (Shao et al., 2017). Pan-Nematoda IntFams also provide a connection that can guide the use of *C. elegans* to investigate intestinal functions with broad application to nematode pathogens.

## The Future of Parasitic Nematode Intestine Research

### Further Development of the *A. suum* Experimental System

The *A. suum* experimental system is well recognized as an important model for parasite research. Creative approaches by many investigators have capitalized on the advantages that this large parasite offers; yet, the potential of the model is far from fully realized. Development of the model for research on intestinal cell biology is at an early stage, with progress driving desire for much more detail, and extensive intestinal omics data will facilitate derivation of many hypotheses. For instance, while longitudinal queries by RNAseq delineate rather gross assessment of differentiation along the intestinal tract, functional nuances among individual intestinal cells interrogated by single cell sequencing (Hwang et al., 2018) remain an intriguing goal to achieve. Major interests of ours include AIM functions and intracellular processes that mediate secretion and renewal of functions that are sited on the AIM. Additionally, more recent findings have implicated exosomal vesicles containing miRNAs derived from the *H. polygyrus* intestine (presumably secreted from the AIM) in modifying host immune responses (Buck et al., 2014; Coakley et al., 2017). Improving the knowledge base and experimental methods for the nematode intestine may contribute to research on the worm side of the interaction. In these contexts, pan-Nematoda omics data coupled with the cannulation/perfusion system offer substantial capabilities to investigate hypotheses related to AIM biology, and efforts are needed to identify the best ways to maintain and manipulate intestinal cells outside of the parasite. Although proteomics data provide valuable information on AIM constituents, this resource can and should be expanded to more comprehensively document functions on the AIM. The adult intestinal cell system will support many experiments; however, the effort and cost to obtain adult *A. suum* are not without drawbacks. Larval stages of *A. suum* (lung stage larvae or early intestinal stages) can provide a less expensive and more ready source of parasite material and are likely to support investigation of some hypotheses on intestinal cells while extending application to additional life cycle stages. Larval stages have generated some useful findings relevant to intestinal cell biology (Wang et al., 2013a). Thus, effort is needed to establish capacity to utilize larval stages for experimental purposes directed at intestinal cell functions. The list can go on, but the areas mentioned are the ones that have priority for us.

### Drug Targets and Therapies

Omics data for intestinal cells and compartments, such as the AIM, provide enormous potential when integrated with drug and target databases to identify prospective parasite targets for existing inhibitors. Areas that are ripe for investigation included enzymes and membrane transporters localized to the AIM. Alternatively, an approach we are following involves predictions of essential cellular pathways and components, which preferentially are pan-Nematoda conserved. One example here involves pathways that transport secretory vesicles containing digestive enzymes and other cargo to the AIM. The goal is to more broadly clarify parasite components that are involved in the exocytic process and identify inhibitors that might replicate irreparable intestinal damage caused by benzimidazole anthelmintics. Whether or not new anthelmintics result, experimentation of this kind is likely to produce new information on basic intestinal cell biology that has potential for pan-Nematoda applications. Further, although the research is intestino-centric, results may have applications to other tissues and cells. Using secretion as an example, secretions in intestinal cells and neurons likely have similar and perhaps identical cellular components, although the consequences of inhibition may be idiosyncratic to a given tissue (tissue degeneration, or disruption of neurotransmission, respectively). There is evidence in *C. elegans* that some proteins that mediate/regulate exocytosis (e.g., syntaxin) have similar functions in each tissue type. Thus, learning more about intestinal cell secretion and inhibitors may have application to other parasite tissues, including the nervous system.

### Vaccine Targets and Immune Control

Development of subunit vaccines against parasitic helminths (with some exception of tapeworms) has proven an elusive goal. Although not without caveats, given the remarkable levels of protection using intestinal antigens against blood-feeding nematodes, progress summarized has identified multiple categories of proteins with potential application to immune control. In addition to likely digestive enzymes, numerous predicted integral membrane and transport proteins were identified that are likely to be localized to the AIM, and host antibody consumed by the parasite would have access to them. Many of these proteins have predicted orthologs in other nematode species, which creates opportunities for research in those species. This dataset, and ideally in the future a larger dataset, identifies numerous functions that can be investigated with antibody-mediated inhibition of those functions. Recognizing that the best immunization results have been achieved thus far in context of blood-feeding parasites, the approach may also have application to somatically migrating stages of parasites such as *A. suum*, which will be in contact with antibodies found in serum. Transcripts for many adult intestinal genes have already been identified, inclusive of those encoding AIM-localized proteins. These genes are expressed in migratory larvae as well, although it remains unclear how extensively these larvae rely on feeding during their migration to the intestine. In any case, the omics data presented stimulate many lines of thought on possible applications of this information to future vaccine development related studies.

## Author Contributions

DJP, RT, and MM all contributed to the writing and all approved the content of this review article.

## Funding

The research outlined in this study was supported by the National Institute of General Medical Sciences Grant R01GM097435 to MM.

## Conflict of Interest Statement

The authors declare that the research was conducted in the absence of any commercial or financial relationships that could be construed as a potential conflict of interest.
